# University students’ satisfaction and future outlook towards forced remote learning during a global pandemic

**DOI:** 10.1186/s40561-022-00197-8

**Published:** 2022-03-21

**Authors:** Siti Intan Nurdiana Wong Abdullah, Klara Arokiyasamy, Sock Leng Goh, Andrea Joveena Culas, Nor Masheera Abdul Manaf

**Affiliations:** Faculty of Business and Communication, INTI International University, Persiaran Perdana BBN, Putra Nilai, 71800 Nilai, Negeri Sembilan Malaysia

**Keywords:** Higher education, Continuous usage intention, Forced remote learning, Pandemic, Satisfaction, Well-being

## Abstract

Technology has enabled the higher education ecosystem to tailor to the students who have diverse needs and to engage with them remotely, especially when face-to-face interaction is not possible. This research contributes knowledge in forced remote learning during the unprecedented global pandemic situation of Covid-19. Using a cross-sectional quantitative method, a total of 480 respondents among undergraduate students from five private universities in Malaysia participated in this study. The data was analysed using structural equation modelling and results indicated that online feedback, online future relevance, online interaction, online teaching effectiveness, and personal well-being were statistically significant in influencing students’ satisfaction. Moreover, online learning satisfaction directly predicted 68.3% of the students’ continuous usage intention while their usage intention was heightened with higher levels of proficiency in online learning. Students’ satisfaction was found to be a significant mediator between all the factors towards usage intention except online assessment, online support, and personal well-being. This study provides the higher education institutions with insights to continuously improve their online delivery strategies and bridge the gap with their students during the pandemic crisis.

## Introduction

Covid-19, a respiratory illness, emerged from the Wuhan province of China in December 2019 and it has since spread to the entire world. World Health Organization (WHO) has reported that the COVID-19 pandemic has spread to 213 countries with a fatality of 2,020,733 globally (WHO, 2020). In Malaysia, the first Covid-19 case was reported on 25 January 2020 and there was an increase in the number by the end of February 2020 due to a mass religious gathering (Tang, 2020). With a persistent increase in new COVID cases, Prime Minister Muhyiddin Yassin rolled out a Movement Control Order (MCO) on 18 March 2020, requiring closure of all businesses except those providing essential services and items. Due to this pandemic and MCO, there was a huge disruption in lifestyles. This has resulted in businesses, including education institutions, to leverage on technology in order to continue operating.

This COVID-19 epidemic has an impact on everyone’s lifestyle including the education sector all over the world (Mustafa, 2020). The COVID-19 had resulted in schools shutting down all across the world, and had over 1.2 billion children out of the classroom (Cathy & Farah, 2020). Many private higher education institutions (PHEIs) had to embrace remote online teaching and learning with the strict MCO in Malaysia. Significant amount of money and time were invested to ensure students are not short-changed in acquiring the related and relevant knowledge for their future and career (Lee, 2010). Large sum of money had to be invested by PHEIs to implement better online learning systems that would encourage students’ usage and reduce attrition rates during the pandemic. Some of these institutions had introduced blended learning and also fully online courses while others set-up the teaching and learning (TNL) unit to train their lecturers to use online learning management platforms such as Blackboard in order for online classes to be conducted seamlessly. Proper guidance and training had to be provided to lecturers in order to ensure the success of the online teaching and learning. Podcasts and tutorials were made available for all teaching staff and students, while ensuring that there was adequate support and guidance for online learning. This transformation had bought both advantages and disadvantages for the students and also the lecturers. Although many universities have used online learning, there still lacks of clear understanding about how students’ experiences could influence their satisfaction and continuance intention.

With the increase popularity of wireless technology applications, online learning platforms become the main solution that provides HEIs broader reach, more convenience, collaboration and customization compared to traditional classrooms (Shiue et al., 2019). Nonetheless, adopting new technology for teaching is challenging to achieve the success of students and educational institutions itself due to the negative attitude and perceptions towards online learning (Al Meajel, 2018; Dhawan, 2020; Hopkins et al, 2020). In addition, a recent study found that students’ attitudes and satisfaction towards remote online learning exerted no influence on intention to continue using this method (Ashrafi et al., 2020). In a traditional classroom setting, factors associated with student satisfaction are often more tangible such as the amenities and facilities provided, quality and qualifications of the lecturers, including support services and activities available (Han et al., 2019; Hsin-i et al., 2021). On the contrary, remote online learning poses diverse challenges to instructors and students especially when implemented under the MCO circumstances which could have put them under tremendous pressure (Guangul et al., 2020; Heng & Sol, 2021). In view of this, HEIs realize the pressing need to overcome the technological obstacles and to be well-equipped for online teaching and learning especially during the pandemic. As students are the main stakeholders at the receiving end, it is necessary to understand the factors affecting their satisfaction (Peterson et al., 2019). Although numerous studies mentioned above may have investigated students’ satisfaction towards online learning, it becomes essential to understand how the current situation could have impacted them.

Taking all of these into consideration, the main objective of this study is to investigate students’ perception on forced remote learning during a global crisis. Judging from the importance of the future trend in online learning and the uncertain situation, it is crucial for private HEIs to understand students’ satisfaction and continuous usage intentions towards online learning as the global pandemic may not be over soon. More specifically, this study aims to determine the key factors influencing students’ online learning satisfaction, and to examine if satisfaction would mediate the relationships between the key factors and students’ continuous usage intention. This study also hopes to fill in the gaps by investigating the moderating role of gender and level of proficiency on the relationship between students’ satisfaction and their intention. Overall, it is hoped that this study will contribute greatly to the development of relevant strategies that will help to improve the effectiveness of remote learning, specifically in the current pressing times**.**

## Literature review and conceptual framework

### Students’ satisfaction

Student satisfaction is defined as student’s feelings of perceived value of the education content and services that they have obtained in return of their time and resources sacrificed (Shahsavar & Sudzina, 2017). Researchers focused on student satisfaction because it is an important outcome that is imperative in influencing students’ motivation and academic performance (Hwang & Choi, 2019). Students who are highly satisfied have been said to be more committed towards online learning as satisfaction could influence their retention and continuous usage intention (Bhattacherjee & Premkumar, 2004; Wu et al., 2017). For instance, satisfaction was confirmed as a key driver of intention to continue use Massive Open Online Courses (MOOCs) (Lu et al., 2019). Despite the research attention on student and lecturers’ readiness to accept new educational technologies, student satisfaction and continuous usage is an equally important consideration.

In order to uncover students’ satisfaction, acceptance and intention towards online learning, past studies have applied various theories such as Technology Acceptance Model (TAM), Unified Theory of Acceptance and Use of Technology (UTAUT), Expectation-Confirmation Theory (ECT), Theory of Planned Behaviour (TPB) and Satisfaction-Loyalty Theory (SLT). This study implores to utilize the ECT and SLT, whereby satisfaction levels would be determined based on students’ experience and perception in online learning that may have exceeded their expectations (Eveleth et al., 2015). According to these theories, students who had a positive perception of their online learning experience would be satisfied and this satisfaction level would then determine their loyalty behaviour, including their intention to continue using the online method of learning. The main rationale for using ECT to explain the students’ satisfaction level is because it emphasizes on post consumption perceptions. Nonetheless, measuring satisfaction is complex as students’ online learning experiences may have been influenced by the current pandemic situation and the quality of education services received.

Many of the past researchers have attempted to examine the private HEIs quality level by focusing on students’ satisfaction (Daud et al., 2019; Hwang & Choi, 2019; Rodrigues et al., 2019). For instance, Latif et al. (2019) developed the HiEduQual to measure service quality in HEIs which includes quality of the educators, institutional leaders, administrative services, knowledge services, university activities, and continuous improvement procedures. Prior to Covid-19, most of the past studies on student satisfaction focused on measuring the tangible factors such as campus facilities, classroom environment, and quality of hardware provided (Daud et al., 2019; Shahsavar & Sudzina, 2017). However, with the recent disruption caused by the Covid-19 pandemic to the education systems and students’ learning trajectories globally, the shift to online learning has placed many HEIs under pressure to adapt quickly (Mishra et al., 2020).

### Factors influencing students’ satisfaction on remote learning

Remote learning has diverse pros and cons. Past research suggests that remote learning has been shown to increase retention of information and takes less time (De Freitas et al., 2015). In addition, remote online learning gives more flexibility to the students and lecturers to learn and work from home. On the other hand, when students encounter problems, they may be too shy to ask questions through the online live classes and some may not be giving their full attention as their lecturers cannot monitor them face to face when the webcams are switched off. These are just a few of the many challenges in remote online learning environment. Further discussions of the key factors influencing their satisfaction towards remote learning are provided below.

In comparison to a traditional campus-based course, online assessments (OAS) were normally designed to be less demanding and lighter (De Freitas et al., 2015). Assessments with clear guidelines and requirements have influenced students’ satisfaction levels that led to their successful completion of a course (Lei & Yin, 2020; Thistoll & Yates, 2016). Students who are clear of what is expected from them tend to have lower stress and anxiety levels. Thus, course assessments should be openly communicated from the start of the course. Ineffective and over-demanding assessments tend to demotivate students while assessments with appropriate level of difficulties had a positive impact on their interest and satisfaction (De Freitas et al., 2015).

For forced remote online learning to be successfully implemented, online feedback (OFB) plays a crucial role as a form of knowledge transfer (Baber, 2020). Instructors are expected to provide timely feedback to the students to keep them engaged. However, overflowing feedback tend to make the students feel overwhelmed while lack of feedback increases the students’ dissatisfaction (Wongwatkit et al., 2020). Feedback on assignments and online activities allows students to know their areas of improvement. From the instructor’s perspective, providing feedback is a form of monitoring students’ progress and ensuring that they complete a course successfully (Roddy et al., 2017). However, OFB proves to be challenging compared to campus-based course since the feedback is not transpired face-to-face with the student and not all the students are open to constructive criticism.

As established in past studies, instructors are the main facilitators and their online teaching effectiveness (OTE) is a main predictor of students’ satisfaction (Glazier & Harris, 2021; Kennette & Redd, 2015; Stickney et al., 2019). Instructors who display the ability to deliver the course content effectively, have a good level of expertise in the subject matter, apply a variety of online tools and can manage their online classroom environment are all critical factors to engage with students (Roddy et al., 2017). Although the capability of academic staffs were the least important criteria in measuring quality of the HEIs, most of the online courses requires the instructors’ presence to effectively deliver the course (Naidu & Derani, 2016). Paechter et al. (2010) found that course design, instructor’s expertise, flexibility, self-motivation, and personal communication skills were also relevant factors in determining students’ overall online learning experience.

In addition to the interactions between the student and instructor, another important aspect of students’ remote learning satisfaction is their online interaction (OIT) with each other. The online learning environment is enhanced and made vibrant when students engage in social interaction and collaboration with their peers. Such open communication usually leads to a positive learning experience despite difficulties in implementing it (Dhawan, 2020). The usage of effective collaborative online tools have been found to increase student satisfaction towards online learning as they become more independent and adaptive to the sudden changes (Guiter and et al., 2021; Mohammed Idris et al., 2021). A lack of feeling connected to faculty has been shown in past research to have a significant negative impact on the student's sense of potential for completion of the online course (Moralista & Oducado, 2020). In the long run, social interaction among students in the online environment creates meaningful dialogues and fosters positive relationships (Keaton & Gilbert, 2020).

As discovered in the earlier review, online support (OSP) is one of the most important factors that impact students’ satisfaction. Students who receive technical support and have sufficient technological resources face lower levels of dissatisfaction (Roff, 2018). Students who have limited internet access or software would feel at a disadvantage. Moreover, it was reported that students preferred HEIs that could provide on-site 24-h online technical support (Elhadary et al., 2020). Institutions that provide an all-rounded online support for the students’ academic journey can ensure that students have a positive learning experience (Roddy et al., 2017). This includes good interaction between students and the instructors, and adequate academic resources such as e-books, videos and other reference materials. Additionally, online students also rely on technological software and hardware to enable them to learn synchronously without any delays or disruptions. They expect that an on-going online technical support is available to them at any time.

Remote online learning draws on the ability of students to learn independently which is consistent with lifelong learning principles (Gibson et al., 2020). Moreover, course design that is linked to real world challenges improves students’ soft skills and increases their employability in the highly competitive job market (De Freitas et al., 2015). Online future relevance (OFR) is described as the level in which students perceive their online course content and activities that would fulfill their personal needs to achieve future desired career goals (Knoster & Myers, 2020). Learning should be meaningful, relevant and interesting though online future relevance has received little scholarly attention (Knoster & Myers, 2020). According to a study by Stoner and Billings (2020) on a pharmaceutical course, alignment of curricular with actual pharmacy practices has improved overall student satisfaction.

According to Roddy et al. (2017), the success in implementing online learning is dependent on four pillars, which are online academic support, technological support, personal well-being and sense of belongingness but these factors were often overlooked. One of the crucial pillars is the personal online well-being (OWB) support provided by education institutions to help students overcome the pressures and anxiety in managing online learning. With the rise of concern on students’ mental health, it becomes a priority to provide counselling and develop preventive strategies (Son et al., 2020). Forced remote learning during the pandemic has been said to cause numerous adverse effects on students’ well-being. Students have noticed changes in their sleeping and eating habits, difficulty in concentrating, deterioration of eyesight due to prolonged usage of computers, feelings of loneliness and panic attacks (Son et al., 2020). Clearly, their negative personal well-being hinders their ability to focus on their studies and decreases their satisfaction levels towards online learning.

Satisfying the needs of large international audiences with diverse knowledge and learning backgrounds is challenging (Gibson et al., 2020). The success of completing a course online strongly depends on the student’s ability to work autonomously and manage their time effectively (Wang et al., 2013). Student’s proficiency in using technology and their perceived satisfaction with online courses is also important to consider if they would be accustomed to continue learning in an online environment (Lee, 2010). Students must have access to reliable equipment and be familiar with the technology used in the course in order to be successful (Belanger & Jordan, 2000). Many HEIs provide orientation for online students to adequately integrate and ease the incoming cohorts into their new online learning environment (Cho, 2012).

Moreover, social influence was the only construct that was found as moderated by gender, where men showed a stronger behavioural intention to use mobile learning technology as opposed to women (Alasmari & Zhang, 2019). In a study on online learning among business students, gender moderated the relationships between performance and system usage whereby female students displayed stronger intentions (Aliyu et al., 2019). This was supported by Alghamdi et al (2020), where they discovered that female students had stronger self regulation behaviors which led to a positive online learning experience, while male students had more stable attitudes towards online learning (Nistor, 2013). Among school students, gender was found to moderate between intention to use e-learning tools and their performance (Wongwatkit et al., 2020). The recent study of Yu (2021) suggested that due to the moderating role of gender in online learning especially during pandemic, teachers should apply different course designs and teaching styles. In Australia, gender moderated between deep learning and satisfaction whereby older female students showed greater levels of deep learning. However, among Millennial students, gender was not found to be moderating online learning satisfaction (Harvey et al., 2017). In another study by Chung et al., (2020) on Malaysian university students’ readiness to use online learning during Covid-19, they found that gender had no significant effect on the overall online learning readiness. Thus, the findings on gender influence in online learning context has been inconclusive so far. Due to the inconsistent findings, it is worth investigating if students’ proficiency and gender plays a moderating role between satisfaction and their intention.

**Fig. 1:**
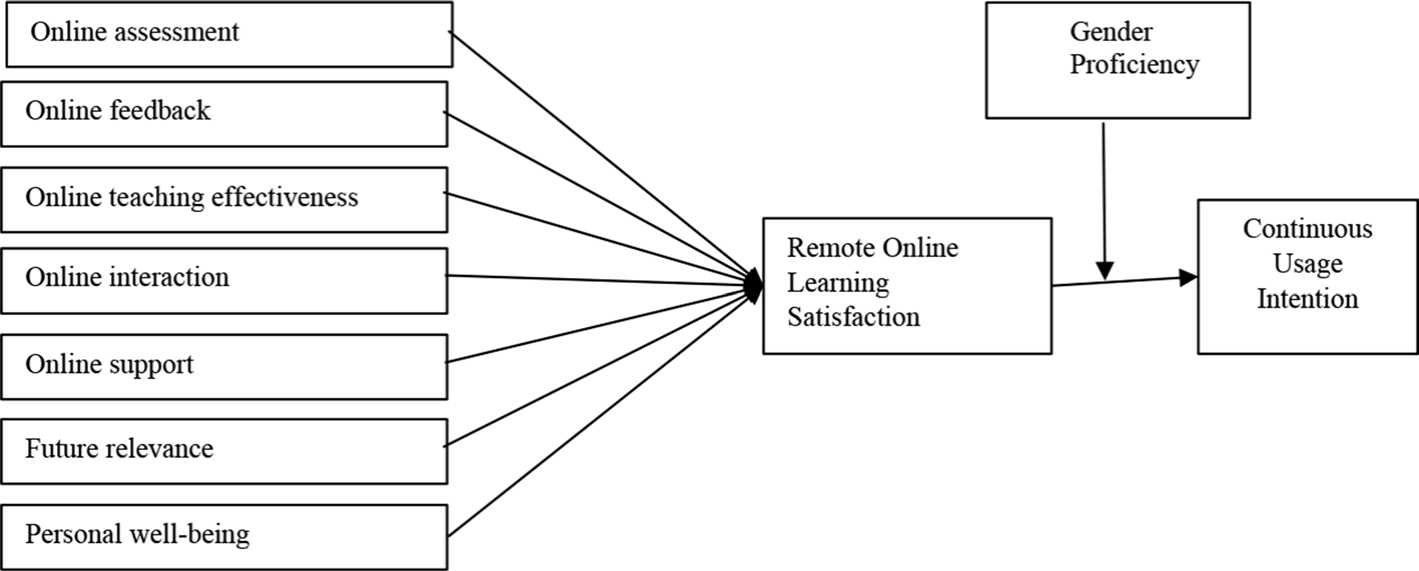
Proposed conceptual framework

Figure 1 shows the proposed framework for this study.

## Methodology

This research was a cross-sectional quantitative study, which utilized survey method. The survey contained three parts, namely Part A- Demographic Background, Part B – Current Online Learning Patterns and Part C – Perception towards Online Learning. The questions in Part A and B used categorical options while the questions in Part C applied a five-point Likert rating scale of strongly agree, agree, neither agree nor disagree, disagree, and strongly disagree.. This study adapted questionnaire items from existing literature. Statements measuring online feedback and online assessment were adapted from Bahati et al., (2019) and Özden et al., (2005); online teaching effectiveness and online interaction from Paechter et al. (2010); online support and personal well-being from Elhadary et al., (2020); and lastly future relevance statements were adapted from Knoster and Myers (2020). For content validity, the research instrument was verified by two experts who are senior lecturers in the area of educational technology and language respectively. Then, the reliability of the research instrument was conducted through a pilot study that involved 30 students from a private university in Malaysia using purposive sampling. It is recommended that a range from 10 to 30 individuals are sufficient for a pilot test using internet survey (Hill, 1998). The overall Cronbach’s alpha was 0.80, above the general rule of thumb of 0.70 which showed that this research instrument has internal consistencies (Nunnally, 1978). For the full data collection, a purposive sampling method was used whereby five lecturers from five different private HEIs located in Malaysia (3 from Peninsular Malaysia and 2 from East Malaysia) distributed the survey to their undergraduate students who has valid registered university email addresses through their respective universities’ online learning platforms (Blackboard and Moodle). The respondents remained anonymous and participated voluntarily in the study. It is advised that the appropriate sample size should be at least 384 (Bujang et al., 2018). Data was gathered using online questionnaire at the end of the semesters for better responses and the students completed the questionnaire in a self-administered manner. A total of 500 questionnaires were distributed and 480 completed questionnaires were returned, giving a response rate of 96%. Descriptive statistics were analysed using SPSS Ver.24, while the structural modelling was then analysed using Smart PLS 3.0 software to confirm the hypothesis. Subsequently, an open-ended semi-structured interview was conducted on six students on a voluntary basis to enrich the quantitative data. A brief introduction and explanation of the purpose of the interview were provided at the beginning, alongside their consent. Namely three questions were asked, *“We have been in the pandemic and have been doing online learning for about 3 years now, what were/are the challenges have you faced?”, “Were there any parts of remote learning that you like or dislike?”, and “Online learning may become a trend in the future, what would you suggest to make online learning better?”*. To ensure anonymity of the participants, their names were not disclosed and only their basic background information were provided. The interviews were recorded and transcribed, then interpreted and discussed alongside the quantitative findings. Each interview ranged between 20 to 30 min and students were asked to provide their honest and truthful opinions to the questions.

### Demographic characteristics

For the demographic profile of the respondents, majority (52.9%) of them were female and 47.1% were male students (Table 1). As for their nationality, we received responses from mainly local students (85.4%) and only 14.6% of them were international students. In terms of their age, majority of 85.2% are between 18 and 22 years old, followed by 9.2% are between 23 and 27 years old; 3.5% are 28 to 32 years old and 2.1% are between 33 and 37 years of age. In Malaysia, there are numerous online undergraduate degree programs that are offered and catered to working adults. To study in a degree program in Malaysia, you need to be at least 18 years old but there is no minimum age requirement for enrolling as long as the entry requirements are fulfilled (Study Malaysia, 2015). Moreover, the flexibility of online learning especially during pandemic has been increasingly popular among school-leavers and some working adults, hence the age gap of 18 to 37 years old. Table 2 shows the demographic breakdown of the six students who participated in the qualitative interview.

**Table 1:** Demographic background of respondents (quantitative survey)

	Frequency	Percentage
Gender		
Female	254	52.9
Male	226	47.1
Nationality		
Local	410	85.4
International	70	14.6
Age		
18–22 years old	409	85.2
23–27 years old	44	9.2
28–32 years old	17	3.5
33–37 years old	10	2.1

**Table 2:** Demographic background of respondents (qualitative interview)

Student number	Gender	Age	Nationality
S1	Female	20	Local
S2	Male	20	Local
S3	Male	20	Foreign (Indonesia)
S4	Female	20	Foreign (Japan)
S5	Female	21	Foreign (China)
S6	Male	27	Local

## Results and discussions

In total, 480 usable responses were received and the descriptive results were obtained using SPSS software. Then, a two-step structural equation modelling using partial least squares method were applied in this study. Firstly, the measurement model is assessed, followed by testing the structural model to determine the results of the hypothesis. All the relevant results are presented in the below subsequent sections.

### Assessment of measurement model

At the measurement model stage, the convergent validity and discriminate validity were assessed according to the criteria suggested by Henseler et al. (2015). Table 3 shows the results for all the convergent validity analysis for the latent constructs which includes the composite reliability, average variance extracted (AVE), square root of AVE, and correlations among constructs. The measurement model demonstrates convergent validity if the factor loadings are above 0.7 (Hair et al., 2019). Items with loadings of 0.6 to 0.7 were retained as the corresponding AVE values were above 0.5 (Ramayah et al., 2018). In this case, the convergent validity is well demonstrated as all the AVE values for the constructs were higher than the suggested threshold value of 0.50 (Gefen & Straub, 2005), while all the composite reliability (CR) results were above the threshold of 0.7 (Sarstedt et al., 2019). The Cronbach’s Alpha (CA) values also met the minimum value of 0.7 as suggested by Hair et al., (2019) and indicated an internal consistency among the measurement items.

**Table 3:** Convergent validity analysis

Factors	Items	Truncated statement	Loadings	AVE	CR	CA
Online assessment (OAS)	OAS1	Clear instructions to complete online assessments	0.809	0.616	0.889	0.844
	OAS2	Virtual consultation hours were useful to complete my assessments	0.788			
	OAS3	Able to complete my online assessments in a timely manner	0.778			
	OAS4	Online assessments enhanced my understanding	0.858			
	OAS5	Completed online assessments with honesty	0.681			
Online Feedback (OFB)	OFB1	Received timely feedback	0.811	0.689	0.917	0.887
	OFB2	Provided constructive feedback for improvement	0.840			
	OFB3	Access to the marking rubrics online	0.834			
	OFB4	Easy access to the online feedback	0.862			
	OFB5	Read all the feedback online	0.803			
Online Teaching Effectiveness (OTE)	OTE1	It is just as effective as F2F teaching	0.759	0.663	0.887	0.833
	OTE2	Variety of online interactive tools while teaching	0.836			
	OTE3	Kept virtual classes lively	0.844			
	OTE4	Virtual classes were more interactive	0.815			
Online Interaction (OIT)	OIT1	Could explain my ideas to others in online discussions	0.789	0.646	0.901	0.862
	OIT2	Interactive online discussions with other students	0.832			
	OIT3	Convenience to discuss group work online	0.848			
	OIT4	Comfortable to communicate with my lecturers online	0.820			
	OIT5	Got to know new friends online	0.724			
Online Support (OSP)	OSP1	Sufficient IT resources for online learning	0.691	0.661	0.906	0.871
	OSP2	Strong internet network access	0.777			
	OSP3	Extra online assistance is provided by the university	0.836			
	OSP4	Sufficient technical support for online learning	0.870			
	OSP5	Easy for me to contact the university	0.876			
Future Relevance (OFR)	OFR1	Online learning made my studies more relevant	0.892	0.754	0.924	0.891
	OFR2	Online materials put me in a real-world context	0.871			
	OFR3	Knowledge gained is relevant to my future career	0.883			
	OFR4	Skills learnt online is necessary for my future job	0.826			
Personal Well-being (OWB)	OWB1	Online workload is too heavy	0.904	0.84	0.954	0.939
	OWB2	Caused me to have difficulty in sleeping	0.938			
	OWB3	Eyesight has deteriorate	0.936			
	OWB4	Online learning makes me nervous	0.886			
Remote Online Learning Satisfaction (OLS)	OLS1	Had a positive online learning experience	0.869	0.756	0.925	0.892
	OLS2	Satisfied with my online learning experience	0.898			
	OLS3	More effective compared to traditional classes	0.852			
	OLS4	Enjoyed online learning more	0.858			
Continuous Usage Intention (CI)	CI1	Happy to continue using online learning method	0.940	0.875	0.954	0.928
	CI2	Would recommend my friends to use online learning method	0.949			
	CI3	Should use online learning as the future direction	0.916			

To assess the discriminant validity, the result of the Fornell–Larcker’s criterion analysis is presented in Table 4. In this study, all the indicators’ loading values (in bold) are higher than the loadings of other constructs. The measurement model demonstrated good discriminant validity since the comparison between the square-root of AVE with the correlations among the constructs indicated that each construct is more closely related to its own measures than other constructs (Hair et al., 2019).

**Table 4:** Fornell–Larcker criterion analysis

	CI	OAS	OFB	OFR	OIT	OLS	OSP	OTE
Continuous Usage Intention (CI)	**0.935**							
Online Assessment (OAS)	0.357	**0.785**						
Online Feedback (OFB)	0.306	0.764	**0.830**					
Online Future Relevance (OFR)	0.651	0.497	0.478	**0.868**				
Online Interaction (OIT)	0.646	0.562	0.509	0.683	**0.804**			
Remote Online Learning Satisfaction (OLS)	0.826	0.480	0.403	0.701	0.741	**0.870**		
Online Support (OSP)	0.531	0.483	0.472	0.774	0.64	0.621	**0.813**	
Online Teaching Effectiveness (OTE)	0.578	0.673	0.615	0.626	0.732	0.681	0.613	**0.814**
Personal Well-being (OWB)	− 0.017	0.037	0.033	0.045	0.015	− 0.035	0.010	0.050

### Assessment of structural model

**Fig. 2:**
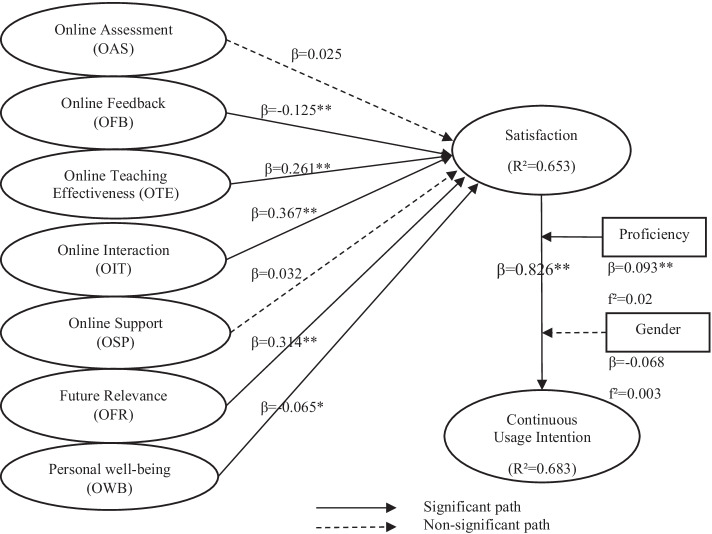
Path coefficient results. Note: *p < 0.05; **p < 0.01

We then assessed the structural model to confirm the proposed hypotheses. Figure 2 shows the results of the hypothesis testing, including the estimated path coefficients and the variance explained (R^2^ value) of the endogenous variables. A bootstrapping (resampling 5000) was conducted as recommended by Hair et al. (2019).

The results of the hypothesis testing are summarized in Table 5. Based on recommendation of Di Leo and Sardanelli (2020), results with lower *p* value of 0.05 can be assumed to be statistically significant, except in medical-related studies. Among a total of seven factors, the direct relationships between satisfaction and five of the factors—online feedback (OFB), online future relevance (OFR), online interaction (OIT), online teaching effectiveness (OTE) and personal well-being (OWB) were all statistically significant (H_a_2 to H_a_4 and H_a_6–H_a_7). The overall coefficient determination of the model indicated that the R^2^ result of 65.3% of online learning satisfaction could be explained by the five factors. The present study confirmed that OIT, OFR and OTE were the top three most important factors that could impact the students’ satisfaction.

**Table 5:** Hypothesis testing results

Hypothesis	Relationship	Path coefficient (β)	SE	t-value	*p* value	Decision
H1	H_o_: OAS has no significant relationship with OLS H_a_: OAS has a significant relationship with OLS	0.025	0.051	0.488	0.313	H_o_ accepted H_a_ rejected
H2	H_o_: OFB has no significant relationship with OLS H_a_: OFB has a significant relationship with OLS	− 0.125	0.041	3.075	0.001	H_o_ rejected H_a_ accepted
H3	H_o_: OTE has no significant relationship with OLS H_a_: OTE has a significant relationship with OLS	0.261	0.052	4.998	0.000	H_o_ rejected H_a_ accepted
H4	H_o_: OIT has no significant relationship with OLS H_a_: OIT has a significant relationship with OLS	0.367	0.052	7.018	0.000	H_o_ rejected H_a_ accepted
H5	H_o_: OSP has no significant relationship with OLS H_a_: OSP has a significant relationship with OLS	0.032	0.048	0.662	0.254	H_o_ accepted H_a_ rejected
H6	H_o_: OFR has no significant relationship with OLS H_a_: OFR has a significant relationship with OLS	0.314	0.055	5.683	0.000	H_o_ rejected H_a_ accepted
H7	H_o_: OWB has no significant relationship with OLS H_a_: OWB has a significant relationship with OLS	− 0.065	0.034	1.936	0.026	H_o_ rejected H_a_ accepted
H8	H_o_: OLS has no significant relationship with CI H_a_: OLS has a significant relationship with CI	0.826	0.015	53.789	0.000	H_o_ rejected H_a_ accepted
H9a	H_o_: OLS does not mediate the relationship between OAS and CI H_a_: OLS does mediate the relationship between OAS and CI	0.021	0.043	0.477	0.634	H_o_ accepted H_a_ rejected
H9b	H_o_: OLS does not mediate the relationship between OFB and CI H_a_: OLS does mediate the relationship between OFB and CI	− 0.103	− 0.101	2.975	0.003	H_o_ rejected H_a_ accepted
H9c	H_o_: OLS does not mediate the relationship between OTE and CI H_a_: OLS does mediate the relationship between OTE and CI	0.216	0.044	4.901	0.000	H_o_ rejected H_a_ accepted
H9d	H_o_: OLS does not mediate the relationship between OIT and CI H_a_: OLS does mediate the relationship between OIT and CI	0.303	0.042	7.228	0.000	H_o_ rejected H_a_ accepted
H9e	H_o_: OLS does not mediate the relationship between OSP and CI H_a_: OLS does mediate the relationship between OSP and CI	0.026	0.041	0.637	0.525	H_o_ accepted H_a_ rejected
H9f	H_o_: OLS does not mediate the relationship between OWB and CI H_a_: OLS does mediate the relationship between OWB and CI	− 0.054	0.029	1.882	0.060	H_o_ accepted H_a_ rejected
H10	H_o_: Gender does not moderate the relationship between OLS and CI H_a_: Gender does moderate the relationship between OLS and CI	− 0.068	0.051	1.340	0.090	H_o_ accepted H_a_ rejected
H11	H_o_: Proficiency does not moderate the relationship between OLS and CI H_a_: Proficiency does moderate the relationship between OLS and CI	0.093	0.030	3.077	0.001	H_o_ rejected H_a_ accepted

If *p* value < 0.05, then H_o_ is rejected and H_a_ is accepted

If *p* value > 0.05, then H_o_ is accepted and H_a_ is rejected

Past research has identified that adequate quality and quantity of interaction between students, their instructor and also peers is associated with increased student course satisfaction (Lee, 2010; Ralston-Berg et al., 2015). This is supported by the responses received from the interview conducted, where most of them mentioned that communication and interaction is the main challenge they face in online learning, regardless of local or foreign students.

*S1: Communication was a challenge. I find it difficult to interact with lecturers, I feel shy to turn on camera and mic to speak.*

*S4: It’s difficult to communicate especially during group assignments, I don’t know my group members.*

*S5: Lack of interaction with lecturers as I’m not able to meet lecturers face-to-face for consultation.*

Moreover, online learning satisfaction directly predicted the students’ continuous usage intention (H_a_8) with the R^2^ of 68.3%. According to Daneji et al., (2019), satisfaction is a strong predictor of continuance usage intention of an online system. Hiltz (1995) found that students with positive attitudes were more satisfied with the online experience and spent more time actively engaged online. Based on the interview, some of the students are satisfied with online learning because of the flexibility, convenience, availability of recorded sessions, reading materials and open book exams.

*S1: Doing revision is easier because I can refer back to the recording. I like the chat option and typing on slide which is convenient.*

*S2: I can save time because I do not need to move physically from one class to another.*

*S3: I like (online class) because I don’t need to carry bag, no need make preparation. Can save time. Flexible, I can eat and drink while having class. For test and exams, I like it because its open book.*

*S6: More convenient, I like lecturers who put information online—I can see anytime. Last time need to print and photocopy, so troublesome.*

The mediation analysis (H_a_9a–H_a_9f) results confirmed that remote online learning satisfaction (OLS) was a significant mediator between all the factors except the relationships of online assessment (OAS), online support (OSP), and personal well-being (OWB) towards continuous usage intention (CI). Past studies have found that the quality and quantity of interaction between a student and their instructor, and student with their peers increases satisfaction (Lee, 2010; Ralston-Berg et al., 2015). Students’ social wellbeing and their academic performance were found to be positively correlated (Samad et al., 2019). Students who are often encouraged to interact with their classmates through online break-out discussion sessions, online group activities and forums to encourage collaboration form a sense of community among themselves (Trespalacios & Uribe-Florez, 2020).

Interestingly, OFB and OWB had a negative influence on their satisfaction. Students’ satisfaction towards forced remote online learning decreases when they felt that their personal well-being is being threatened. From the interview, one of the students (S4) stated that she doesn’t like online learning because her eyes get tired from using the computer for too long. In hindsight, long usage of computers has been said to lead to various health issues, such as deteriorating eyesight and carpal tunnel syndrome (Ellahi et al., 2011). Past literature found that 50% of university students felt a certain level of mental stress when enrolled in online programs (Regehr et al., 2013). Moreover, the personal well-being of the students is not really known by the HEIs due to the physical distance, making the provision of mental and physiological support really challenging (Wrench et al., 2014) especially when these students were not given a choice in view of the pandemic situation.

Contrary to the hypothesized relationships, students’ learning satisfaction (OLS) was not influenced by online assessment (OAS) and online support (OSP), thus rejecting H_a_1 and H_a_5. From the interview, only one student faced an internet connectivity issue—“*I face internet connection problems, so I cannot hear what the lecturer is explaining. I couldn’t answer when lecturer asking question, I can’t follow”.* The students who face absence of internet access at home may feel that this problem may not be under the control of the university, but rather their own choice of internet service provider. Beyond the traditional classroom, private HEIs have indeed invested in its technological infrastructure and online helpdesk that enabled them to assist students who faced any technical issues. Private HEIs often emphasized on high quality services for their students even before the global pandemic started (Jalali & Islam, 2011). The online support continued to pour in to convince students that regardless of the current situation, their access to education must be continued. Moreover, online method of assessing students have already been practiced in most of the private HEIs whereby students have submitted their assignments and conducted their presentations through online learning platforms. Hence, the students were unfazed by these two factors. However, these factors may become more relevant if final assessments were to be conducted and monitored fully online, it may become more critical for students to feel that they are still being fully supported remotely.

**Fig. 3:**
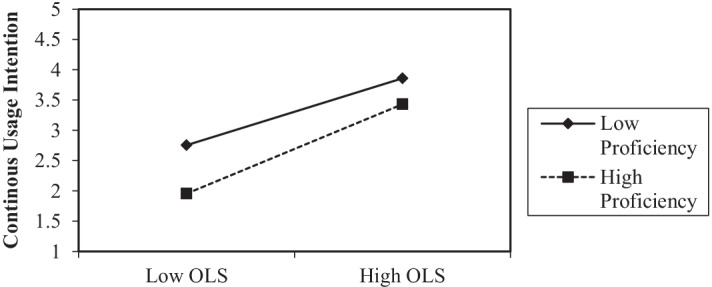
Interaction plots of moderating effect

Another key finding of this study is the moderating roles of gender and the students’ level of proficiency (H10). To test this hypothesis, the product-indicator approach was applied. As can be observed from the result depicted in Table 5, the interaction term of OLS*Gender is not significant despite some of the earlier studies having confirmed male students to be demonstrating higher computer self-efficacy than female students (He & Freeman, 2010; Karsten & Schmidt, 2008). Whereas, to test for the moderating effect of proficiency, the orthogonalization approach is used for continuous variable as suggested by Fassott et al., (2016). The interactions of OLS*Proficiency are also found to be positively significant (t-value = 3.077; *p* value = 0.001) and the effect size f^2^ of 0.02 was considered to be small as indicated by Cohen (1988). Thus, H_a_11 is accepted and the interaction plot shown in Fig. 3 indicates that the positive relationship between satisfaction and continuous usage intention is stronger when the students’ level of proficiency in online learning is higher. Noteworthy, the success of the forced remote online learning is found to be highly dependent on university students’ continued usage (Lee, 2010).

## Conclusion and recommendations

Understanding the forced remote learning environment from students’ perspective is important for higher education in current pandemic situation. Along that line, this research has made several contributions to existing literature on remote learning and teaching in general. In this research, we focused on university students in private HEIs. Firstly, this research systematically and empirically examined the impacts of various key factors from students’ perspectives towards forced remote learning. The conceptual model was developed to assess the impacts of online assessments, online feedback, online interaction, online teaching effectiveness, online support, online future relevance, and personal well-being of students towards their satisfaction and continuous usage intention. Surprisingly, online assessment and online support were confirmed to have no significant influence on students’ learning satisfaction. In view of this, students may have been unaffected by the online assessments if clear guidelines were already provided by the instructor. Moreover, most of the HEIs in Malaysia have yet to fully depend on online assessments for the final examinations. Many of the private HEIs in Malaysia have implemented alternative methods of assessing the students instead, such as using take-home assignments and group projects to replace final examinations. Assessments for online learning have also been said to be less burdensome compared to traditional campus-based exams (De Freitas et al., 2015). As for the insignificant role of online support, it is noteworthy that majority of this study’s respondents are local students (85.4%). Although there is still room for improvements in the network infrastructure, Malaysia’s inclusive internet index currently ranks 8^th^ among 35 counties in the Asia–Pacific region (The Star, 2020). This study also evaluated the moderating role of gender and proficiency level of students towards the relationships between satisfaction and their intention. Interestingly, our findings discovered that gender had no influence while students with higher level of proficiency did show stronger intention to continue learning online.

This study also has certain limitations that future research can undertake to provide more in-depth findings. The current study only focused on a few private universities in Malaysia and respondents drawn were mainly local students. In future, this study can be expanded to other public and private universities with more balanced responses from both local and international students in order to provide a better overview of their satisfaction and usage intention of online learning. Moreover, a comparative study can be conducted to determine the differences in their satisfaction between traditional face-to-face, fully online learning, and blended learning pedagogy. Additionally, future studies could also probe more deeply into students’ mental and physical health, their coping mechanisms and other social context factors (such as family members and friends) that could influence their current online learning experiences. Lastly, course design could also play an important role in determining the students’ satisfaction levels. Hence, future studies should consider a breakdown of respondents by their course design, program, age groups or cohort that could account for the differences in learning patterns and their satisfaction towards lecturers and total satisfaction of online learning.

As remote online learning continues to develop during the Covid-19 pandemic, necessary adjustments need to be made by HEIs to ensure that these courses remain relevant and beneficial for students, and to limit the disruption to the entire education system. With the move of many HEIs in offering online courses, there is an increased responsibility to understand how technology can be harnessed to provide students with the best learning experiences, and to better prepare them for the changing future needs. Based on this study, below are some of the recommended strategies for HEIs to consider implementing to enhance their online learning ecosystem.

### Encourage interaction

Although COVID-19 pandemic has made society, HEIs, lecturers and students gradually accept the online class mode and become more and more familiar with the online operation, it also offers great opportunities for instructors to explore other interactive tools to be applied in their classes. For example, a simple, easy-to-use digital online learning application page is the start, but embedded within the course, instructors can use other tools like Padlet to capture live comments from students and Kaltura to create their own video mash-up of a topic. According to the students interviewed, they suggested that lecturers could interact with students on a non-academic basis to build rapport.

*S5: I think the lecturer's language should be designed with a sense of dialogue, avoiding long monologues and leaving us time to think. The lecturer can also be interactive, so that we are more motivated to learn.*

*S6: Add more activities between lecturers and students (non academic activities would be good). Lecturers can share their own stories to make it more interesting.*

This is also supported by Tang and Hew (2017), whereby they concluded that online learning platforms are equivalent to a community site or social network sites like Twitter that thrives on interactions. Instructors could also take a step back during the small group break-out sessions to allow students to have a more open communication with each other while working simultaneously on a shared file like Google Jamboard or Canvas App. Some of the students interviewed also felt that engagement can be improved if students switch on their cameras.

*S2: Need to ensure people turn on their cameras to ensure better engagement.*

*S4: I think it’s good to open camera for 1st week of class, so we can familiarize with each other.*

#### Up-to-date online content and design

The role and responsibilities of education has never ceased to be important in a country’s development. Today, employers are becoming more concerned with what they call the “skills gap” in graduates. Hence, HEIs can encourage more business organisations from various sectors to provide inputs during the course design and other learning opportunities for students such as through employer projects. In hindsight, education providers should forge partnerships with employers and even government agencies to facilitate updated online content and successful work-integrated learning opportunities for the students. Instructors should devise the course content and methods of assessment that are appropriate to students’ professional development and future career goals.

### Well-being support for both lecturers and students

Due to the intensive remote online learning and given the added pressures both students and instructors are facing what with shorter course deadlines and being physically segregated from their peers, HEIs are required to implement appropriate prevention and intervention strategies to overcome the high rates of distress. As students and lecturers go through the transitioning period, HEIs can provide personalized counselling sessions to deal with their mental health issues. Some of the students may become disengaged and suffer in silence as a form of self-protection mechanism. Hence, providing sufficient support system is important to recognize any symptoms of depression. As for the lecturers, they may be suffering from burnout and stress from juggling between work demands and family needs. The management of HEIs must avoid micromanaging their employees and strike a balance by not being over-demanding with the achievements of organizational goals. Besides, one of the students (S3) interviewed suggested that internet quota can be provided for certain students by the universities. He also recommends that students computers’ be installed with speech-to-text software that acts as subtitles when lecturers are conducting the online classes. For instance, IBM Watson or Google Speech.

### Relevant and timely feedback using analytics

It is recommended that students need to be provided with regular feedback to ensure their remote online learning process runs smoothly. With the use of built-in analytics in most of the online learning platforms like Blackboard and Moodle, instructors can monitor the students’ online presence and reach out to them. In most circumstances, students are expecting feedback to be sufficient, timely and personalized. It was confirmed by Lim et al., (2019) that students displayed higher self-discipline and performed better academically when they received feedback compared to those who didn’t. However, in using analytics in these platforms to provide feedback, it should be just as a tool to capture relevant data for early intervention. Instructors should not fully rely on the learning analytics as a form of punishment or negative feedbacks. Some consideration should be taken on students’ emotions. Feedback should be communicated carefully to students who would reciprocate in a positive manner.

## Data Availability

This research data is available upon request via email to the corresponding author.

## Abbreviations

AVE: Average Variance Extracted
CA: Cronbach’s Alpha
CR: Composite Reliability
CI: Continuous Usage Intention
ECT: Expectation-Confirmation Theory
HEI: Higher Education Institution
MCO: Movement Control Order
MOOC: Massive Open Online Courses
OAS: Online Assessments
OFB: Online Feedback
OFR: Online Future Relevance
OIT: Online Interaction
OLS: Remote Online Learning Satisfaction
OSP: Online Support
OTE: Online Teaching Effectiveness
OWB: Online Personal Well-being
PHEI: Private Higher Education Institution
SLT: Satisfaction-Loyalty Theory
TAM: Technology Acceptance Model
TNL: Teaching and Learning
TPB: Theory of Planned Behaviour
UTAUT: Unified Theory of Acceptance and Use of Technology
WHO: World Health Organization
